# Online monitoring of the mitochondrial respiration activity and protein formation in the Almost Living Cell-free Expression (ALiCE) system

**DOI:** 10.1186/s12896-025-01029-6

**Published:** 2025-08-30

**Authors:** Paul-Joachim Niehoff, Sarah Luise Straaten, Anna Luca Ida Hampe, Yannick Flaskamp, Johannes Hemmerich, Hannes Juergens, Robin Roentgen, Ricarda Finnern, Jochen Büchs

**Affiliations:** 1 Chair of Biochemical Engineering (AVT.BioVT), RWTH Aachen University, 52074 Aachen, Germany; 2 LenioBio GmbH, 40231 Düsseldorf, Germany

**Keywords:** Cell-free protein synthesis, CFPS, ALiCE, Oxygen transfer rate, eYFP

## Abstract

**Background:**

Cell-free protein synthesis (CFPS) is one approach to address the increasing demand for complex recombinant proteins in various applications, especially in the pharmaceutical sector. CFPS offers a variety of advantages like the ability to express cytotoxic proteins, no need for transformations or screening of strains and, thus, reduced production times. Often industrially relevant proteins require post-translational modifications (PTM). While disulfide bonds can be obtained with prokaryotic systems, some eukaryotic CFPS systems are able to perform glycosylation to a limited extent. However, scaling the production of a eukaryotic CFPS system and protein production has been a main challenge to enable the manufacturing of complex proteins with these CFPSs. One plant-based system that overcomes these limitations is the Almost Living Cell-free Expression (ALiCE) system, which is based on tobacco BY-2 cells and can produce protein titers of up to 3 µg/µL in batch mode. This study focuses on gaining a deeper understanding of oxygen demand, protein formation and the role of mitochondria in this CFPS system.

**Results:**

Online monitoring was established in a combined µRAMOS-BioLector device to investigate the correlation of oxygen transfer, eYFP production and NADH levels during the ALiCE reaction. By varying the maximum oxygen transfer capacity, it was revealed that oxygen availability is tightly coupled to protein formation and that the eYFP production rate decreases with decreasing oxygen availability. Moreover, the mitochondrial inhibitors salicylhydroxamic acid (SHAM) and potassium cyanide (KCN) were added to ALiCE reactions to examine the influence and importance of mitochondrial alternative oxidase and cytochrome c oxidase on the ALiCE reaction. Inhibition of alternative oxidase and cytochrome c oxidase demonstrated that oxygen is consumed in the respiratory chain of intact mitochondria within the ALiCE system. In addition, the NADH balance and eYFP formation are highly dependent on oxygen availability.

**Conclusions:**

For the first time, a plant-based cell-free expression system was characterized concerning oxygen demand and the influence of oxygen availability on the kinetics of protein production. The new findings enable the design of ALiCE experiments in mL-scale with optimal oxygen supply for protein formation in the future and provide first insights into the energy metabolism of this plant-based CFPS system.

**Supplementary Information:**

The online version contains supplementary material available at 10.1186/s12896-025-01029-6.

## Background

Nowadays, more than 170 complex, recombinant proteins are used worldwide in medicine, for example, components of vaccines and diagnostics [1]. The demand for such complex proteins and, therefore, also the need for rapid and efficient screening and production platforms is further increasing. Cell-free protein synthesis (CFPS) is one key component to address this demand [2]. First introduced in 1961 [3], CFPS systems have undergone many improvements and tackled challenges that in vivo expression systems face. They show a multitude of advantages compared to cell-based systems. These include the ability to produce difficult-to-express or cytotoxic proteins [4–6] and to reduce production times since there is no need for transformations or screening of strains [7, 8]. However, the cost of CFPS systems is often higher as energy components or free amino acids must be supplemented to the lysate [9]. On the other hand, nonstandard amino acids can be incorporated, leading to a wider range of proteins [10]. Furthermore, the reaction conditions can easily be adjusted to facilitate protein expression and folding [11–13]. These advantages lead to a broad application range from therapeutic proteins [14, 15] over high-throughput screening platforms [16, 17] to the construction of metabolic pathways [18, 19] or biosensors [20, 21]. Even though CFPS are showing huge potential in these fields, their currently limited scalability prevents their application for large-scale production purposes.

CFPS platforms based on many different organisms were developed depending on the desired application. CFPS systems based on *Escherichia coli* or wheat germ extract are widely used, but there is also research focusing on other prokaryotes and yeasts like the fast-growing *Vibrio natriegens* [22, 23], members of *Bacillus* sp. [24, 25] or *Pichia pastoris* [26, 27]. The advantage of these systems is their fast production time and high protein titers. The highest titer for *E. coli* based CFPS platforms has been reported to reach up to 2.3 g/L in batch mode [28], while *V. natriegens* lysate produced up to 1.6 g/L protein in batch mode [29]. However, these platforms are unsuited for most proteins, needing complex post-translational modifications (PTM) like glycosylation. New technologies like bacterial glycoengineering [30, 31] have shown huge potential to overcome these limitations. For more complex PTMs, platforms based on plant [32–34], insect [35, 36], or mammalian cells can be used [37–41]. However, the protein yield is lower for most of these systems than in bacterial systems [42]. Tobacco cell-based platforms circumvent this challenge, showing high protein titers, a relatively low preparation time and the ability to perform PTM [43]. The Almost Living Cell-free Expression (ALiCE) system is based on tobacco BY-2 cells and can produce complex proteins with a high product titer of up to 3 µg/µL in batch mode [34, 43–45]. Even transmembrane proteins can be functionally expressed [46]. However, to date, most experiments were performed in µL or mL-scale with this system. Only one publication showed production of eYFP in L-scale [44]. Hence, scaled production of more complex proteins still has to be shown with ALiCE.

One of the features that sets the ALiCE system apart is the presence of intact organelles, which enables more complex cellular processes [44]. In particular, intact mitochondria provide energy equivalents for transcription and translation [47]. This is possible via the mitochondrial respiratory chain, where ATP is generated under oxygen consumption. Therefore, unlike in established cell-free expression systems, less expensive energy substrates must be added to ALiCE [47, 48].

It has been shown that in-vivo production of proteins is tightly coupled to the availability of oxygen and the respiration activity of the organism [49, 50]. In living cells, a metabolic burden caused by protein production is sometimes reflected in the growth behavior which can be monitored via the respiratory activity using the oxygen transfer rate [51]. However, the respiratory activity of CFPS systems has, to our knowledge, not yet been investigated. In this work, the µ-Respiratory Activity MOnitoring System (µRAMOS) [52] combined with the BioLector technology [53–55] was used to implement online analytics of the ALiCE CFPS reaction. With this device, the oxygen transfer rate can be measured through optical fibers in the headspace of the wells, while e.g. fluorescence can be measured in parallel through the transparent bottom (compare Figure S1). Enhanced yellow fluorescent protein (eYFP) was used as a model protein and measured online with the BioLector technology [53–55]. To allow for reproducibility, these online measurements were compared to the commercial protocol given by LenioBio GmbH. In a second step, the dependency of the ALiCE reaction on oxygen availability was investigated. Lastly, experiments with routinely used mitochondrial inhibitors KCN and SHAM [56–58] were performed to confirm the presence of intact mitochondria and investigate their contribution to the target protein production.

## Results and discussion

### Establishing online monitoring for the lysate reaction

**Fig. 1:**
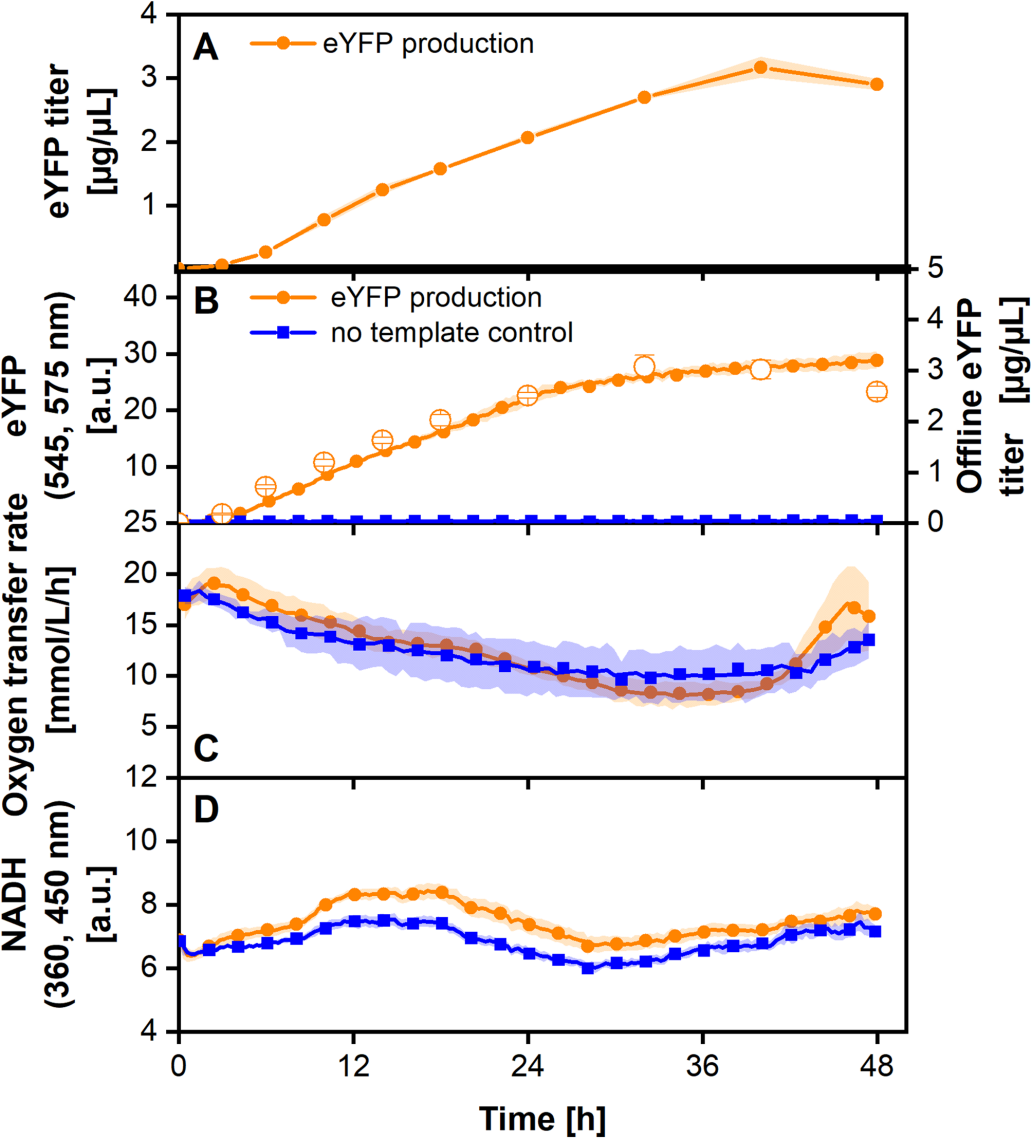
**ALiCE reaction in two types of microtiter plates with simultaneous online monitoring of the oxygen transfer rate (OTR), eYFP and NADH fluorescence.** The lysate reaction (Batch LYCDAV11SC) was carried out (**A**) according to the commercial protocol in a 96 half-area plate with 50 µL filling volume per well, operated at 500 rpm, 12.5 mm shaking diameter and 25 °C and (**B-D**) in a 48-round well plate, 300 µL filling volume per well, operated at 700 rpm, 3 mm shaking diameter and 25 °C in a combined µRAMOS*-*BioLector device. The data was obtained in *N* = 1 for the lysate reaction in the 96 half-area plate, 3 for the no template control and 4 for the lysate reaction in the 48*-*round well plate. Shadows represent the standard deviation of reactions for *N* ≥ 3 and the standard deviation of the technical triplicate measurement for *N* = 1. In parallel, offline-determined eYFP titers are symbolized by empty circles in **B**. Error bars show the standard deviation of *N* = 3. For clear data representation, only every fourth or eighth measurement point is shown for the OTR and eYFP/NADH, respectively

To examine the respiration activity of the ALiCE system, online monitoring had to be established. Simultaneous monitoring of the oxygen consumption and the eYFP formation was achieved by using a combined µRAMOS-BioLector device. In a first step, the behavior of the ALiCE lysate reaction was compared between the combined µRAMOS-BioLector device and the commercial ALiCE reaction protocol (Fig. 1).

The sampled and offline measured eYFP concentration for the commercial ALiCE reaction protocol with 50 µL reaction volume showed an almost linear increase from 3 to 40 h (Fig. 1A). After 40 h, a maximum eYFP titer of 3.2 ± 0.2 µg/µL was reached before it slightly declined to 2.9 ± 0.1 µg/µL at the end of the reaction time. Comparison with a second lysate batch (Figure S2) showed that the general course of eYFP formation over time is reproducible, although the plateau phase differs. However, despite different courses over time a titer of at least 2 µg/µL is achieved and lies within the given specifications by LenioBio.

Comparing the commercial reaction protocol (Fig. 1A) to the offline measured concentrations from the reaction in 48-round well plates in the combined µRAMOS-BioLector device (Fig. 1B), a similar behavior was observed in both reaction vessels. In the 48-round well plates, the offline determined eYFP titer also increased after 3 h. The highest eYFP titer of 3.1 ± 0.2 µg/µL was reached after 32 h, 8 h earlier than using the commercial reaction protocol (Fig. 1A). Afterwards, it decreased to 2.6 ± 0.1 µg/µL.

The difference in the eYFP course over time between the commercial reaction protocol (500 rpm, 12.5 mm shaking diameter, 50 µL in 96 half-area plates) and the reaction in the combined µRAMOS-BioLector device (700 rpm, 3 mm shaking diameter, 300 µL filling volume in 48-round well plates) can likely be attributed to oxygen availability. For the reaction in 48-round well plates at 700 rpm with 300 µL filling volume the correlation by Lattermann et al. (2014) can be used to calculate the OTR_max_ and resulted in 36.3 mmol/L/h. It must be noted that the correlation by Lattermann et al. (2014) does not include the shaking diameter as it was kept constant at 3 mm in the study. Besides using Lattermann’s correlation, a calibration curve with an *E. coli* culture was recorded (Figure S3), to determine the OTR_max_ values for different filling volumes in the combined µRAMOS-BioLector device. For 700 rpm with 300 µL filling volume in 48-round well plates, the determined OTR_max_ is 30 mmol/L/h, which is slightly lower than the 36.3 mmol/L/h from Lattermann et al. (2014). In Fig. 1C, an OTR of around 17 mmol/L/h was measured for the ALiCE reaction at 700 rpm in 48-round well plates (3 mm shaking diameter, 300 µL filling volume) meaning that this reaction was oxygen unlimited as the OTR is lower than the OTR_max_ (36.3 or 30 mmol/L/h respectively). Even though, no online measurement of the oxygen transfer rate is possible in the commercial setup (500 rpm, 12.5 mm shaking diameter, 50 µL in 96 half-area plates) a correlation by Klöckner et al. (2012) was used to estimate the OTR_max_ and resulted in 8.3 mmol/L/h. With oxygen unlimited conditions (Fig. 1C) the ALiCE reaction reached 17 mmol/L/h indicating that the reaction at 500 rpm (12.5 mm shaking diameter with 50 µL filling volume in 96 half-area plates) was oxygen limited as the OTR of ALiCE is higher than the OTR_max_ of 8.3 mmol/L/h. The lower OTR_max_ in 96 half-area plates could be an explanation for the slightly different eYFP production curve (compare Fig. 1A and B), where at 500 rpm (Fig. 1A), the plateau is only reached at around 40 h. However, the OTR_max_ correlation by Klöckner et al. (2012) gives only an estimation and was not verified in the scope of this study.

The online-measured eYFP signal in the 48-round well plate (Fig. 1B) correlated well with the offline-measured eYFP. A linear increase until 26 h, followed by a plateau, was observed in the online signal, while the offline measured concentrations showed a slight degradation at the end of the reaction. The difference between the online and offline measurements was likely due to the manual execution of the assay for the offline determination of eYFP concentrations. No eYFP increase was visible for the no template control (NTC), which does not contain the plasmid with the eYFP gene.

The OTR of the ALiCE reaction did not show the exponential increase normally seen in microbial cultures [59]. In contrast, the OTR started at a high level of 17 mmol/L/h, indicating the high activity of the concentrated protein production machinery in the ALiCE system after thawing. After the start of the experiment, a small increase in the OTR was visible until a peak of 19 mmol/L/h was reached after 2.5 h. This increase was likely due to the necessary time for the ALiCE reaction to reach full metabolic activity. After the peak, the OTR steadily decreased. Possible explanations for the decrease of oxygen consumption are the deterioration of the relevant protein machinery, especially in the mitochondrial respiratory chain, the accumulation of reaction (by-) products leading to a general decrease in reaction rates, or the depletion of energy substrates. The latter seemed most unlikely because a sharp decrease in the OTR would be expected once energy substrates are fully consumed. Nevertheless, a secondary substrate limitation might lead to the observed OTR profile [59, 60]. An exponential increase in OTR was observed after 40 h, likely due to microbial contamination, indicating that substrates suitable for aerobic respiration were still present at this time.

Comparing the NTC to the lysate reaction producing eYFP, the OTR appeared to be very similar. The OTR of the NTC also started at ~ 17 mmol/L/h and then decreased. Between 22 and 42 h, a relatively constant oxygen consumption of 10.5 mmol/L/h was observed. One might expect that protein production would lead to a higher demand for ATP and, therefore, oxygen compared to the NTC. However, oxygen requirements for the fast production of the eYFP protein were approximated at an OTR of around 0.738 mmol/L/h (only translation-related ATP costs considered, see methods section for calculation). More complex proteins would most likely not change this result, as they are often produced at lower rates compared to eYFP. Larger proteins often have more complex folding patterns, which contribute to slower overall synthesis times [61]. In addition, the ALiCE lysate reaction represents a plethora of metabolic pathways and molecular interactions, which, compared to living cells, are not contained within a closed cell membrane and, due to a lack of cellular homeostasis, might be present in non-physiological concentrations [43, 62]. Effects due to macromolecular crowding [63, 64] or regulation through membrane domains [65] are most likely different in this environment. It is conceivable that some metabolic reactions, therefore, run at non-physiological rates, which, in combination with potentially inactive inhibition mechanisms, could lead to a consistently maximal ATP demand and oxygen consumption.

In contrast to the OTR, a difference for the NADH signals could be observed. During the entire reaction time, the NADH signal of the reaction producing eYFP was higher than that of the NTC. Four replicates were run for the NTC and eYFP producing reaction and the standard deviations are very small. During the relevant eYFP production time frame (3 to 24 h) a clear difference between the NADH signals with and without eYFP production is visible. This might be due to a shift in redox metabolism. The eYFP production in the reaction with added plasmid causes a higher ATP demand for e.g. transcription and translation. This higher ATP demand, in turn, requires more NADH which leads to an increase in NADH-generating reactions, e.g. glycolysis or amino acid metabolism.

The data clearly show that the lysate reaction performed similarly in the online monitoring system compared to the commercial reaction protocol in 96 half-area plates. However, online monitoring of the OTR, eYFP production and even NADH gives additional insights into the ALiCE reaction.

### Oxygen availability determines the kinetics of the lysate reaction

**Fig. 2:**
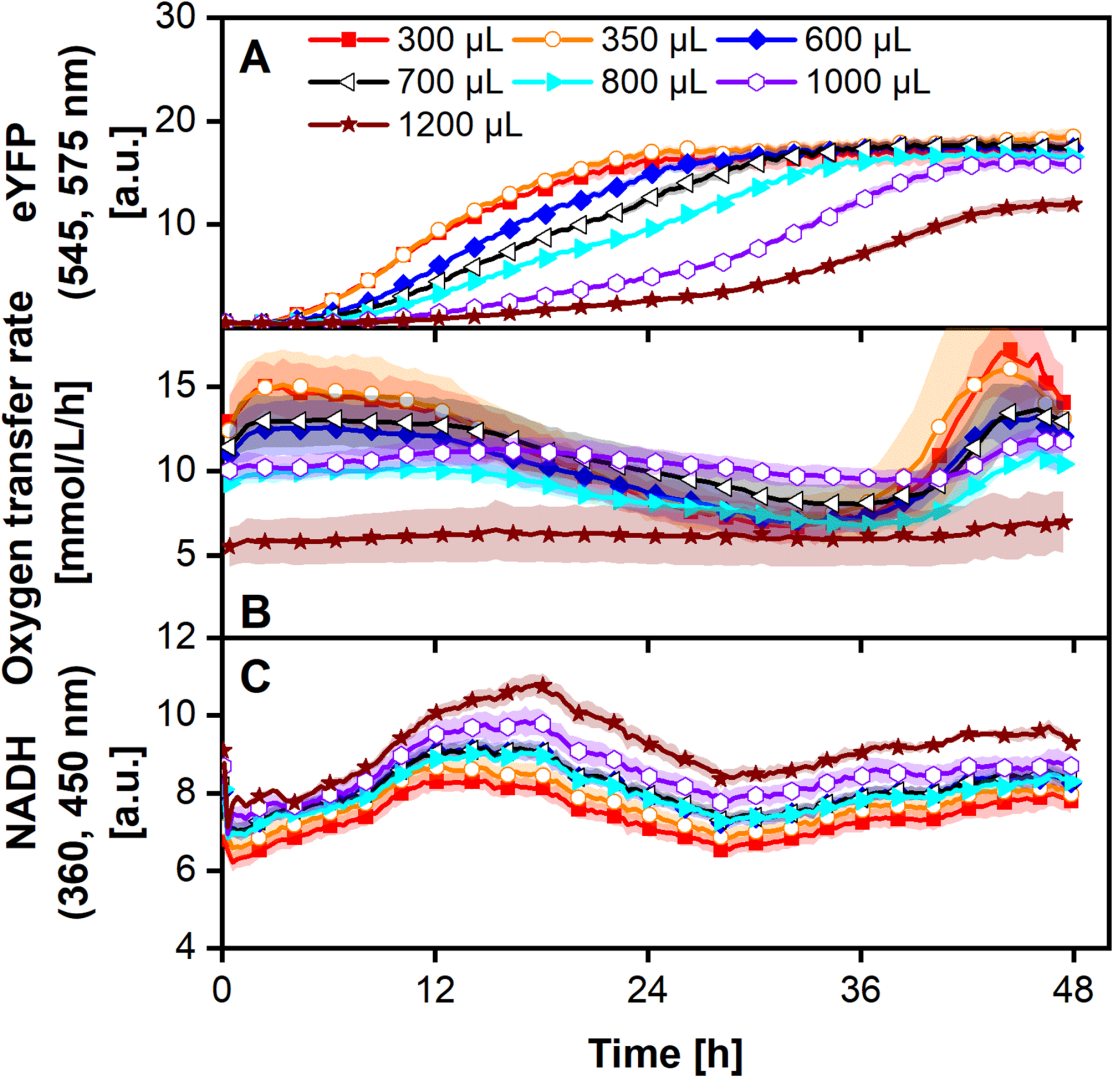
**ALiCE reaction in a 48-round well plate with different filling volumes and online monitoring of the oxygen transfer rate (OTR), eYFP and NADH fluorescence.** The lysate reaction (Batch LYCDBK111SC) was carried out in a 48-round well plate, with 300–1200 µL filling volume per well operated at 700 rpm, 3 mm shaking diameter and 25 °C in a combined µRAMOS-BioLector device. The introduced air was humidified using two washing bottles filled with deionised water. The mean of *N* = 4 replicates is shown. Shadows represent the standard deviation. For clear data representation, only every fourth or eighth measurement point is shown for the OTR and eYFP/NADH fluorescence, respectively. Data for 400 and 500 µL filling volume was omitted for clarity and is shown in the supplementary data (Figure S4)

After successfully establishing the ALiCE reaction in an online-monitored system, the influence of oxygen on the reaction kinetics was further investigated. Thus, an experiment with varying degrees of oxygen availability was conducted (Fig. 2). Here, the filling volume of the wells was varied to reach different maximal oxygen transfer capacities as demonstrated before [55, 66].

Varying the filling volume to change the maximum oxygen transfer capacity of the reaction system may introduce secondary effects, like changes in mixing times, relative evaporation and hydromechanical stress. Under the used conditions, mixing time in shaken bioreactors can be considered very fast with all filling volumes and, thus, be neglected [67]. Evaporation is only limited by the sterile barrier [68] and was measured to be less than 5% for all filling volumes (data not shown) and, thus, also assumed to be negligible. The power input (P) for all filling volumes is the same, as the shaking parameters are kept constant (*n* = 700 rpm, d = 3 mm). However, the volume specific power input PV is highest for the smallest filling volume [69]. Because no negative influence is seen in the experiments with the lowest filling volume, hydromechanical stress is considered to be negligible under the chosen experimental conditions.

ALiCE reactions with 300 and 350 µL filling volume showed an increase in the eYFP signal after 2 h and saturation after 21.5 h (Fig. 2A, Figure S5). As the filling volume increased, the eYFP production was slowed down but reached the same final fluorescence levels, except 1200 µL. The OTR for all filling volumes, except the highest, showed a continuous decrease over time for the first 36 h of the experiment (Fig. 2B). The decrease in the OTR has been observed in a previous experiment (Fig. 1C), with the decrease generally occurring later the higher the filling volume. Potential explanations include the slower deterioration of the mitochondrial electron transport chain and/or slower substrate depletion/byproduct accumulation at lower reaction rates caused by lower oxygen availability.

After an increasing OTR within the first three hours, a plateau was visible for filling volumes of 600 and 700 µL, indicating an oxygen limitation (Fig. 2B, Figure S6) [60]. In addition, the *E. coli* calibration curve for OTR_max_ values (compare Figure S3) was used to evaluate oxygen limitation. The derived OTR_max_ from Figure S3 for 600 µL, is 16 mmol/L/h. As Fig. 1C showed, the ALiCE reaction reaches an OTR of 17–20 mmol/L/h at oxygen unlimited conditions, which is higher than the OTR_max_ of 16 mmol/L/h at 600 µL filling volume. Hence, 600 µL is the first filling volume where an oxygen limitation would be expected and was observed (Fig. 2B, Figure S6). This limitation was even stronger for higher filling volumes, leading to a lower OTR from the beginning of the experiment. The NADH signals supported this hypothesis (Fig. 2C). While the signal’s general pattern was the same for all filling volumes, higher NADH signals could be observed with increasing filling volume, presumably due to the lower availability of oxygen causing lower respiratory activity, leading to an accumulation of NADH.

In general, the eYFP production rate decreased with decreasing oxygen availability. As eYFP requires oxygen for fluorescence maturation [70], this effect should be considered when evaluating the formation under oxygen-limited conditions (e.g. 600 µL). The ALiCE reaction with 600 µL filling volume shows a constant increase in the eYFP signal while the OTR drops around 12 h (Figure S6) as the lysate functionality is probably reduced over time. As the oxygen demand of the metabolic activity of the lysate decreases, more oxygen is available for maturation (increase in dissolved oxygen tension (DOT)). The DOT can be calculated by DOT = 100 *(1 - OTR/OTR_max_) (compare Supplement 10). As OTR_max_ values were determined by the *E. coli* calibration curve (Figure S3), the DOT is above zero, once the OTR is lower than the determined OTR_max_. If maturation of accumulated produced eYFP would occur, the online eYFP signal would increase abruptly [55, 71]. However, the slope of eYFP increase stays the same, indicating that maturation does not influence the online signal significantly. A similar effect of reduced production rates with lower oxygen has been observed before for bacterial CFPS systems, indicating an active respiratory chain [72, 73].

Total eYFP titer was constant for a wide range of filling volumes (300–800 µL) and only decreased at strong oxygen limitations (1000–1200 µL). Once substrates for either protein production (amino acids), transcription, translation, or ATP production are fully consumed or not regenerated, eYFP production stops. A possible explanation for the lower final concentration at 1200 µL is the consumption of the necessary substrate(s) by a microbial contamination. The ALiCE reaction was prepared only with sterile and RNase free tips, but not in a sterile environment. This could cause the contamination indicated by an increase in the OTR at the end of the reactions. Even though there is no increase in the OTR of 1200 µL visible, this is probably masked by the oxygen limitation. Circumventing contamination by using a clean bench during the preparation of the lysate reaction, did not yield any product, likely due to the air stream within the bench and the resulting RNase activity (data not shown).

All in all, most likely the lower oxygen availability results in lower metabolic throughput through the respiratory chain and, thus, in less energy in the form of ATP and an increase in NADH (Fig. 2C). This then leads to slower and - with strong oxygen limitation - lower eYFP formation. To further understand the underlying mechanism, spiking experiments with oxygen and ATP could be performed in future experiments.

**Fig. 3:**
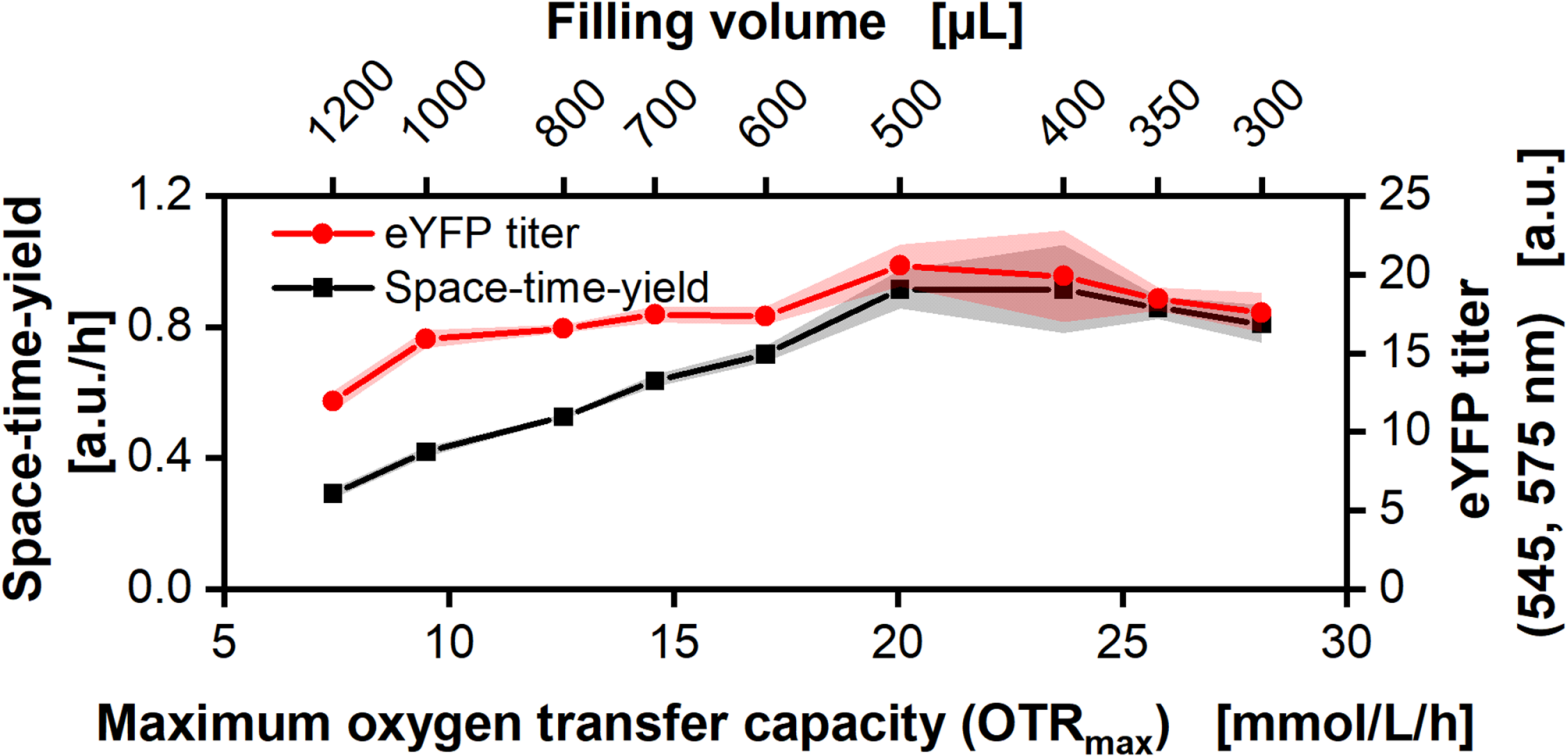
**Correlation of eYFP titer and space-time-yield and maximum oxygen transfer capacity (OTR**_**max**_**).** The maximum oxygen transfer capacity (OTR_max_) was calculated based on the correlation shown in Figure S3, B. The ALiCE CFPS reaction (Batch LYCDBK111SC) was carried out in a 48-round well MTP, with 300–1200 µL filling volume per well operated at 700 rpm, 3 mm shaking diameter and 25 °C in a combined µRAMOS-BioLector device. The mean of *N* = 4 replicates is shown. Shadows represent the standard deviation (compare Fig. 2). “a.u.” = artificial units

Figure 3 shows the final eYFP titer and the space-time-yield (STY) over the maximum oxygen transfer capacity (OTR_max_) for the different filling volumes. The STY showed a linear increase with increasing OTR_max_ up to 20 mmol/L/h before decreasing slightly. While the STY more than doubled from 0.42 a.u./h at an OTR_max_ of 9 mmol/L/h to 0.91 a.u./h at 20 mmol/L/h, the titer only increased from 16 to 20 a.u. The titer decreased abruptly for OTR_max_ lower than 7.5 mmol/L/h, while the STY continued decreasing linearly.

Without oxygen limitation, the ALiCE reaction worked at its maximum capacity and maximum speed (compare Figs. 1 and 2). This led to the highest titer and STY due to the high availability of ATP from the functional respiratory chain. Possibly, a fixed percentage of the produced ATP is available for protein production. The absolute amount of ATP per time, however, got smaller, the stronger the oxygen limitation, explaining the reduced production rates and space-time-yield.

In total, the data showed that with decreasing oxygen availability, the production rate of eYFP decreased linearly. At the same time, the overall titer was only slightly influenced by oxygen limitation for the small range of filling volumes (300–1200 µL) tested in this setup. However, at an OTR_max_ below 7.5 mmol/L/h a strong drop in eYFP titer was observed (corresponding to 1200 µL). Further research in the kinetics of the ALiCE CFPS reaction is required to fully understand the mechanisms underlying this effect.

### Intact mitochondria are responsible for respiratory activity

Mitochondrial inhibition experiments were performed to further analyze respiration activity and determine the source of the oxygen consumption of the ALiCE system.

In plant cells, usually, two types of oxygen-reducing enzymes are present in the mitochondrial respiratory chain: alternative oxidase (AOX) and proton-pumping cytochrome C oxidase (complex IV) [74–76]. AOX is responsible for homeostasis of the energy metabolism [77] and stress reduction of reactive oxygen species (ROS) [76]. Utilization of AOX removes electrons from the respiratory chain before they reach the proton-pumping complexes III and IV, thereby reducing the amount of proton translocation across the inner mitochondrial membrane and reducing the ATP yield per NADH that is re-oxidized by the respiratory chain [77]. As ALiCE contains intact organelles for energy regeneration and protein production, it was hypothesized that the observed oxygen transfer rates in the range of 17 mmol/L/h result from active mitochondria and a functional respiratory chain. Furthermore, the aim was to identify how the two oxygen-reducing mitochondrial enzymes contribute to the respiratory activity and to what extent they contribute to the oxygen consumption of ALiCE and the eYFP production. To this end, AliCE reactions were characterized under the influence of the mitochondrial inhibitors SHAM and KCN, which inhibit AOX and complex IV, respectively [78].

**Fig. 4:**
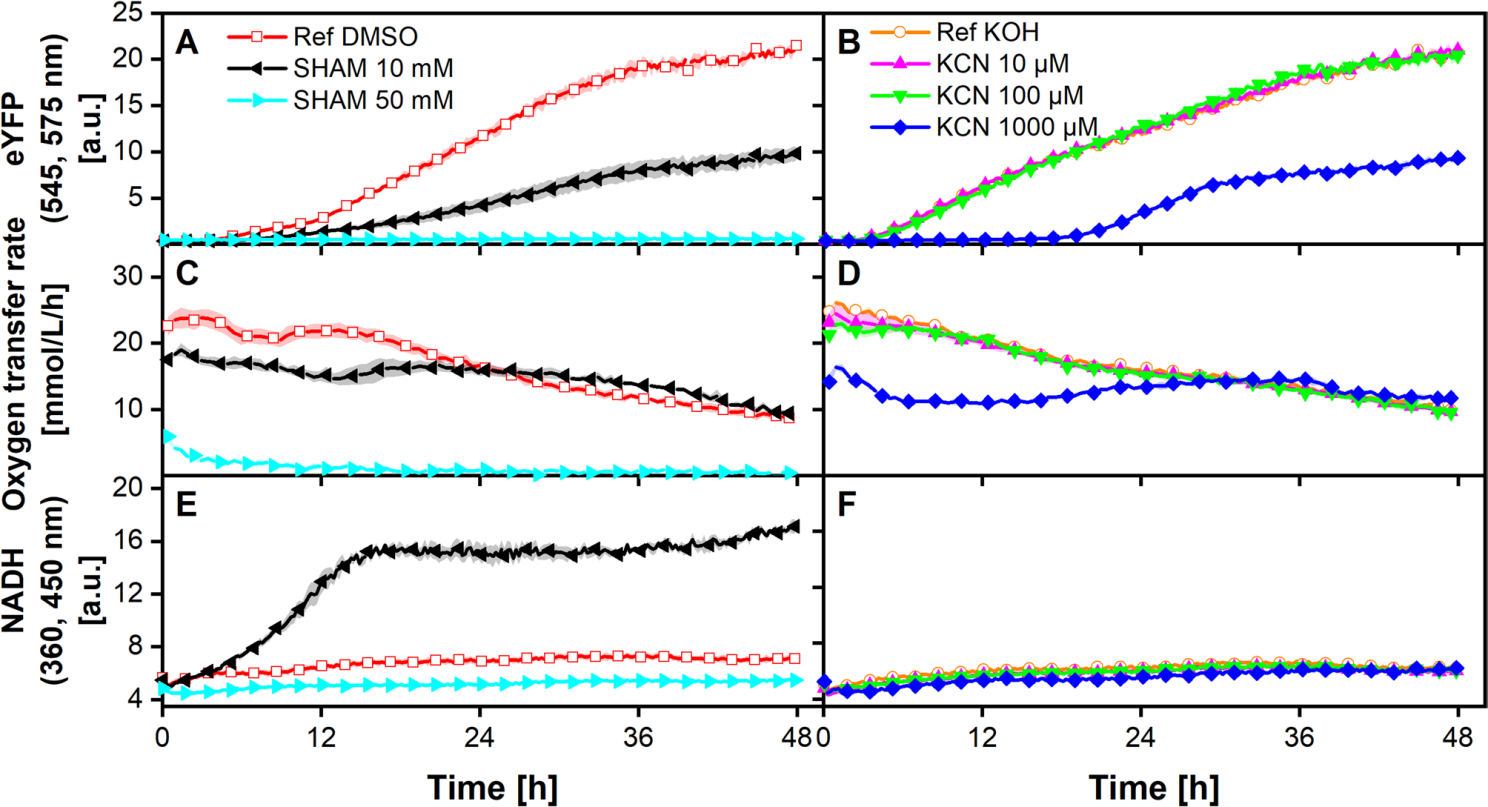
**ALiCE reaction with respiratory inhibitors in a 48-round well plate with online monitoring of the oxygen transfer rate (OTR), eYFP and NADH fluorescence.** The ALiCE CFPS reaction (Batch LYCCCQ11SR) was carried out in a 48-round well plate, 300 µL filling volume per well operated at 700 rpm, 3 mm shaking diameter and 25 °C in a combined µRAMOS-BioLector device. Panels **A**, **C** and **E** show the respective course of eYFP, OTR and NADH for the addition of 10 and 50 mM SHAM and the DMSO (solvent) reference. Panels **B**, **D** and **F** represent the course of eYFP, OTR and NADH for the addition of 10, 100 and 1000 µM KCN and the KOH (solvent) reference. The mean of *N* = 3 replicates is shown. Shadows represent the standard deviation. Due to the high reproducibility of the measurements, the standard deviation might not be visible for every data point. For clear data representation, only every fourth or eighth measurement point is shown for the OTR and eYFP/NADH, respectively

Figure 4 (A, C, E) shows the online data (eYFP, OTR and NADH) for ALiCE reactions supplemented with 10 and 50 mM SHAM and a DMSO reference. For the DMSO reference, the AliCE reaction showed a constant eYFP production rate and reached a maximum protein titer after 36 h (Fig. 4A), similar to previous experiments without DMSO addition. Likewise, the ALiCE reaction exhibited an initial respiratory activity (OTR of 22 mmol/L/h), similar to the reactions without DMSO and a constant NADH signal (Fig. 4C, E respectively). By adding 10 mM SHAM, eYFP production slowed down and final titer was reduced by around 60%, whereas at 50 mM SHAM no eYFP production occurred. With 10 mM SHAM, the ALiCE reactions also showed reduced respiratory activity with an OTR of 19 mmol/L/h, whereas for 50 mM SHAM, only 5 mmol/L/h were measured before oxygen uptake of ALiCE stopped completely (Fig. 4C). The general pattern of a slow decrease in the respiratory activity of ALiCE, starting from 24 h, was observed for the addition of 10 mM SHAM as well as the reference. At 10 mM SHAM, the ALiCE reaction showed high NADH accumulation (Fig. 4E).

Figure 4 (B, D, F) displays the online data, where concentrations between 10 and 1000 µM KCN were tested in the ALiCE reaction. As before, the reference with KOH, which was used as a solvent for KCN, showed the previously established course of reaction for eYFP, OTR and NADH. Reduced respiratory activity of ALiCE was measured only in the first 6 h for 10 and 100 µM KCN, where OTR was lowered by 2–3 mmol/L/h. Afterwards, eYFP formation, OTR, and NADH progress were identical to the KOH reference. When 1000 µM KCN was applied to the ALiCE reaction, inhibitory effects became evident. From 0 to 18 h, the ALiCE reaction showed no eYFP production and a reduced OTR of 12–13 mmol/L/h. After 30 h, inhibitory effects declined, and the respiration level was similar to the ALiCE reactions with lower KCN concentrations. Simultaneously, eYFP formation began and increased in the last 20 h of the reaction to around 50% of the final eYFP titer, compared to the reference reaction. The level of NADH was not affected.

By evaluating the results from both inhibitors, insights into the respiratory characteristics of ALiCE can be gained. Partial inhibition of AOX (10 mM SHAM) seems to sufficiently perturb normal mitochondrial function, strongly shifting the NADH balance and reducing respiratory capacity. Under these conditions, the eYFP formation rate is also negatively affected. Stronger inhibition (50 mM SHAM) abolishes respiratory activity, indicating that AOX fulfills the essential plant cell function of balancing mitochondrial metabolic homeostasis also in ALiCE. In addition, it can be speculated that an accumulation of ROS occurs, which has been observed in studies with plant root cells during AOX inhibition [63]. ROS accumulation could contribute to the observed detrimental effects of AOX inhibition.

Regarding complex IV, the addition of 10 and 100 µM KCN did not result in an observable inhibitory effect, indicating that complex IV from *Nicotiana tabacum* is not affected by this concentration range when present in ALiCE. 1000 µM KCN did elicit an inhibitory effect, which does not appear to be permanent, as eYFP formation is only temporarily halted. The temporary inhibition can likely be attributed to the evaporation of HCN from the experimental setup, which is documented in the literature [79]. Below a pH of 8, which is the case for the ALiCE reaction, the introduced cyanide is mostly present in the form of HCN [64], which can evaporate from the reaction liquid. Inhibition of complex IV by cyanide is reversible [65] so the function of previously inhibited complex IV was likely reinstated as cyanide concentration decreased. The observed abolished eYFP formation due to complex IV inhibition during the first third of the reaction with 1000 µM KCN is consistent with the expected strongly reduced ATP availability under these conditions, as proton-translocation by complexes III and IV is probably affected. The reduction of oxygen consumption to half of the reference value indicates that complex IV is either not completely inhibited or that AOX contributes significantly to oxygen consumption under these conditions.

In summary, we speculate that sufficient activity of both terminal oxidases, AOX and complex IV, is important for normal protein formation in ALiCE. Future research into the interplay of the two enzymes and their relevance for the ALiCE reaction will likely require a more detailed characterization of the effect of mitochondrial inhibitors, including orthogonal validation methods like genetic perturbations [80] or metabolomics [81].

## Conclusion

Online monitoring of the respiration activity and eYFP production of the ALiCE system was established using the in-house built combined µRAMOS-BioLector device. The eYFP signal, oxygen transfer rates and NADH levels were measured online in 48-round well plates and compared to the commercial ALiCE protocol in 96 half-area plates. Both experimental setups showed a similar eYFP formation kinetic. Moreover, a high respiratory activity of the ALiCE system was revealed. It was shown that the kinetics of the reaction are determined by oxygen availability. Based on these findings, a maximum oxygen transfer capacity of at least 20 mmol/L/h was found to be optimal for a high space-time-yield. Furthermore, the importance of a working mitochondrial respiratory chain for protein production in the ALiCE CFPS reaction was demonstrated. Next to working oxygen consumption via complex IV, functional AOX was shown to be essential for maintaining the redox balance and maximizing protein titer.

All in all, in this study, a plant-based cell-free expression system was characterized for the first time by online monitoring concerning oxygen demand, the influence of oxygen availability and respiratory chain function on the kinetics of eYFP production. Both limiting the oxygen availability for the ALiCE reaction via sub-optimal reaction conditions and inhibiting mitochondrial respiration, lead to lower oxygen consumption rates (indicated by the lower measured oxygen transfer rates) and lower protein production rates. Both mechanisms of this effect likely share the same cause, decreased ATP availability from the respiratory chain, underlining its importance for the ALiCE CFPS reaction. Further research is needed to deepen the understanding of redox balances, distribution of metabolic energy, and possible deterioration of the ALiCE system. In addition, the translation of more complex proteins and the scale-up to L-reactions needs to be done to showcase the potential of the ALiCE CFPS system for production purposes.

Understanding the dependency of protein production on oxygen in ALiCE is an important aspect for the design of future experiments. When scaling up the ALiCE reaction, a constant OTR_max_ of 20 mmol/L/h is a suitable scale-up parameter. Maintaining the shaken bioreactor principle, orbitally shaken bioreactors could be used. The k_L_a correlation by Klöckner et al. (2013) demonstrated that orbitally shaken bioreactors with reactor volumes up to 200 L could be used with a filling volume of 40 L (20% relative filling volume) at 100 rpm shaking frequency to achieve an OTR_max_ of 20 mmol/L/h [82]. If higher liquid volumes or a higher relative filling volume is desired, aerated stirred tank reactors would be an option. As the oxygen content of the inlet air can be elevated, higher OTR_max_ values are possible. However, other aspects like hydromechanical stress and mixing are important to investigate and will be subject of future research.

## Materials and methods

### ALiCE reactions

The ALiCE reaction mix and pLB001 plasmid (coding for eYFP) were provided by LenioBio GmbH (Düsseldorf, Germany) and stored at -80 °C and − 20 °C, respectively. The ALiCE batches were produced using a controlled production process with fixed manufacturing instructions and quality control testing. This included reaching at least 3 µg/µL eYFP titer after 48 h of incubation time in reference conditions. For the cell-free protein expression, 2 mL ALiCE tubes (50 to 500 µL filling volume) were thawed in water at room temperature. According to the commercial protocol provided by LenioBio GmbH, 50 µL ALiCE reactions were conducted in 96 half-area plates (Greiner Bio-One GmbH, Frickenhausen, Germany) at 25 °C, 500 rpm, a shaking diameter of 12.5 mm and 80% relative humidity. For online measurements, 300 to 1200 µL ALiCE reactions were carried out in 48-round well plates (Beckman Coulter GmbH, Krefeld, Germany), at 25 °C, 700 rpm, and a shaking diameter of 3 mm. The introduced air was humidified using two washing bottles filled with deionised water. The 96 half-area plates and 48-round well plates were sealed with a polyolefin sealing foil (HJ-Bioanalytik GmbH, Erkelenz, Germany) to reduce evaporation. Thus, the evaporation was determined to be less than 5% and considered negligible (data not shown). The supplemented, final plasmid concentration in the ALiCE reaction was 10 ng/µL for all experiments. The number of replicates is indicated in the caption of the respective experiment.

### Online monitoring

For ALiCE reactions at 48-round well MTP scale, an in-house built µRAMOS device, in combination with the BioLector technology, was used [52, 55, compare Figure S1]. Above every individual well, oxygen-sensitive fluorescence spots determine the oxygen partial pressure (pO2) of the gas phase, using the Stern–Volmer equation for quenching. The OTR was determined as described by Flitsch et al. [52]. eYFP formation was measured online in the combined µRAMOS-BioLector system, with an excitation wavelength of 545 nm and an emission wavelength of 575 nm. At wavelengths 485 nm, 525 nm close to the emission and excitation maximum of eYFP (513 nm, 527 nm), interference with the background matrix was observed, leading to a non-representative eYFP signal over time (compare Figure S7). To facilitate data interpretation, the mentioned excitation and emission wavelength shifted from the maxima were used. An excitation wavelength of 360 nm and an emission wavelength of 450 nm were set for monitoring NADH.

### Offline product analysis

For eYFP offline measurements, a Multi-Detection Microplate Reader (Synergy 4, BioTek, VT, USA) was used. From each biological sample, four technical replicates were measured. The excitation wavelength was 485 nm and the emission wavelength 528 nm. Samples were diluted 1:10 in a 96 half-area plate. The quantity of eYFP was determined by generating a standard curve with different concentrations of purified eYFP with a known concentration (compare Figure S8). The eYFP standard was produced by recycling the eYFP from previous ALiCE reactions, which is fused to a Strep-tag. It was purified using a Strep-Tactin^®^ XT 4Flow column (IBA Lifesciences, Göttingen, Germany), a gravity flow column for Strep-tag fusion proteins. The concentration of purified eYFP was determined by recalibration with a known standard. For offline measurements of samples from the combined µRAMOS-BioLector device, a separate MTP was incubated with the same incubation settings from which samples were taken. For every offline sample, a new, unsampled MTP well was used.

### Determination of space-time-yield and maximal oxygen transfer rate

To determine the space-time-yield (STY) of the ALiCE reactions, the mean of the last five measurements of the eYFP [a.u.] for each filling volume was divided by the time, at which 85% of the maximum eYFP value was reached. This method was chosen to minimize evaporation effects on the eYFP fluorescence intensity (compare Figure S9). The mean of the maximum and the time when 85% of this maximum was reached are shown in Table 1.

**Table 1:** Maximum eYFP signal and times, when 85% are reached, for the calculation of the space-time-yield (STY)

Filling volume [µL]	Maximum eYFP signal [a.u.]	Time 85% of maximum reached [h]
300	17.6	21.7
350	18.4	21.5
400	19.9	21.7
500	20.6	22.5
600	17.4	24.2
700	17.5	27.5
800	16.6	31.5
1000	15.9	38.0
1200	11.9	41.0

Even though a correlation for the maximum oxygen transfer capacity (OTR_max_) for 48-round well plates exists [83], the calculated value (15.1 mmol/L/h) overestimated the clear limitation observed with 1200 µL in this work (~ 6 mmol/L/h, compare Fig. 2). Therefore, a new correlation of the OTR_max_ and the filling volume was created using an *E. coli* BL21 (DE3) pet-Xal-YFP culture in Wilms-MOPS medium, grown at the same conditions as the lysate reaction (48-round well plate, 700 rpm, 3 mm shaking diameter, 25 °C, varying filling volumes) (Figure S3, A). The OTR_max_ was determined from the resulting OTR plateaus [60] and then plotted against the filling volume (Figure S3, B). The resulting calibration curve was used to extrapolate the OTR_max_ of the different filling volumes of the lysate reaction.

### Approximation of required OTR for eYFP production

The approximation of the required OTR for eYFP production was performed by calculating the necessary ATP amount for translation based on the amino acid sequence of eYFP and an estimated P/O ratio of 1.5 [84]. The calculation steps are described in the following:

One molecule of eYFP contains 239 amino acids and has a molecular weight of 27 kDa [85]. The number of required ATP equivalents for each translation step was considered with amino acid activation/tRNA charging: 2 ATP equivalents, initiation: 1 ATP equivalent, elongation: 2 ATP equivalents for each step, and termination: 1 ATP equivalent [86]. For 239 amino acids, this results in 956 ATP equivalents per eYFP molecule. A P/O ratio of 1.5 was assumed, which resulted in 318 O_2_ molecules for 956 ATP and, therefore, one molecule of eYFP. A final titer of 3 µg/µL in 300 µL reaction volume corresponds to 0.9 mg eYFP in total, which was then converted in kDa, so that the respective amount of eYFP molecules and, therefore, O_2_ molecules could be calculated. A total amount of 1.062 × 10^− 5^ mol oxygen was calculated. Considering the reaction time of 48 h and the reaction volume of 300 µL, a required OTR of 0.738 mmol/L/h is estimated.

### Respiratory inhibitors

All chemicals used to inhibit the respiration activity were purchased from Sigma-Aldrich (St. Louis, MO, USA). A 1 M stock solution of salicylhydroxamic acid (SHAM) was prepared in dimethyl sulfoxide (DMSO), while a 0.3 M potassium cyanide (KCN) stock solution was prepared in 100 mM KOH. DMSO and 100 mM KOH without inhibitors were used as references, to investigate only the effect of the inhibitor, respectively.

## Supplementary Information

Below is the link to the electronic supplementary material.

Supplementary Material 1

## Data Availability

The datasets supporting the conclusions of this article are included within the paper and the additional file (Figures S1 – S9, Supplement S10). The datasets used and analyzed during the study are available from the corresponding author upon reasonable request.

## Abbreviations

ALiCE: ALmost Living Cell-free Expression
(µ)RAMOS: (µ) Respiration Activity Monitoring System
a.u: arbitrary units
AOX: Alternative oxidase
ATP: Adenosine triphosphate
CFPS: Cell-free protein synthesis
DMSO: Dimethyl sulfoxide
eYFP: enhanced Yellow Fluorescent Protein
MTP: Microtiter plate
NADH: Nicotinamide adenine dinucleotide
NTC: No template control
OTR: Oxygen transfer rate
OTR_max_: Maximum oxygen transfer capacity
PTM: Post translational modification
ROS: Reactive oxygen species
SHAM: Salicylhydroxamic acid
STY: Space-time-yield
