# Supplementary material for: Online monitoring of the mitochondrial respiration activity and protein formation in the Almost Living Cell-free Expression (ALiCE) system
Source: BMC Biotechnol. 2025 Aug 30;25:93. doi: 10.1186/s12896-025-01029-6 (PMC12398971; doi:10.1186/s12896-025-01029-6)
Supplement: Supplementary file 1 — Supplementary Material 1 [file 12896_2025_1029_MOESM1_ESM.docx]

# Supplementary information


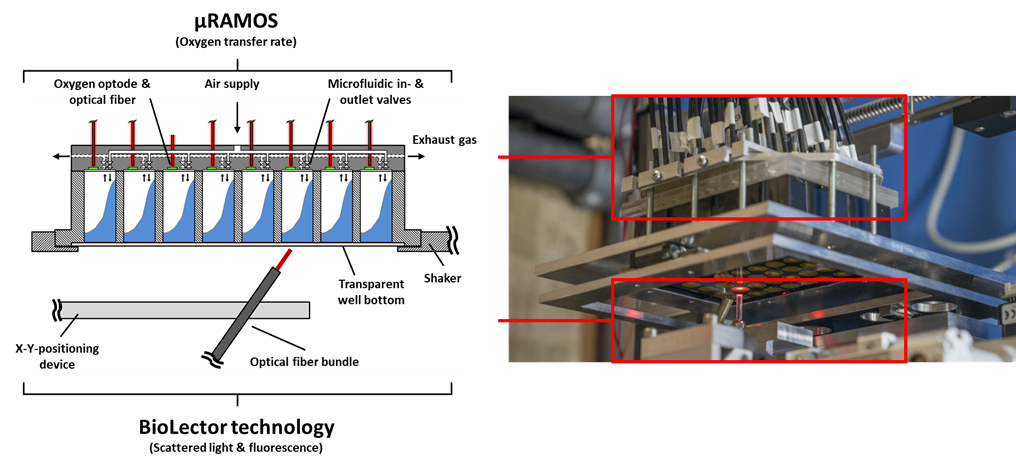


**Figure S1: Schematic illustration of the combined µRAMOS-BioLector device.** Different fluorescence signals and scattered light are monitored for each well of the 48-round well plate through the transparent bottom (BioLector technology (53-55)). By means of the μRAMOS device, the oxygen concentration in the headspace of each well is monitored and the oxygen transfer rate (OTR) is determined. Adapted from Ladner et al. (55).


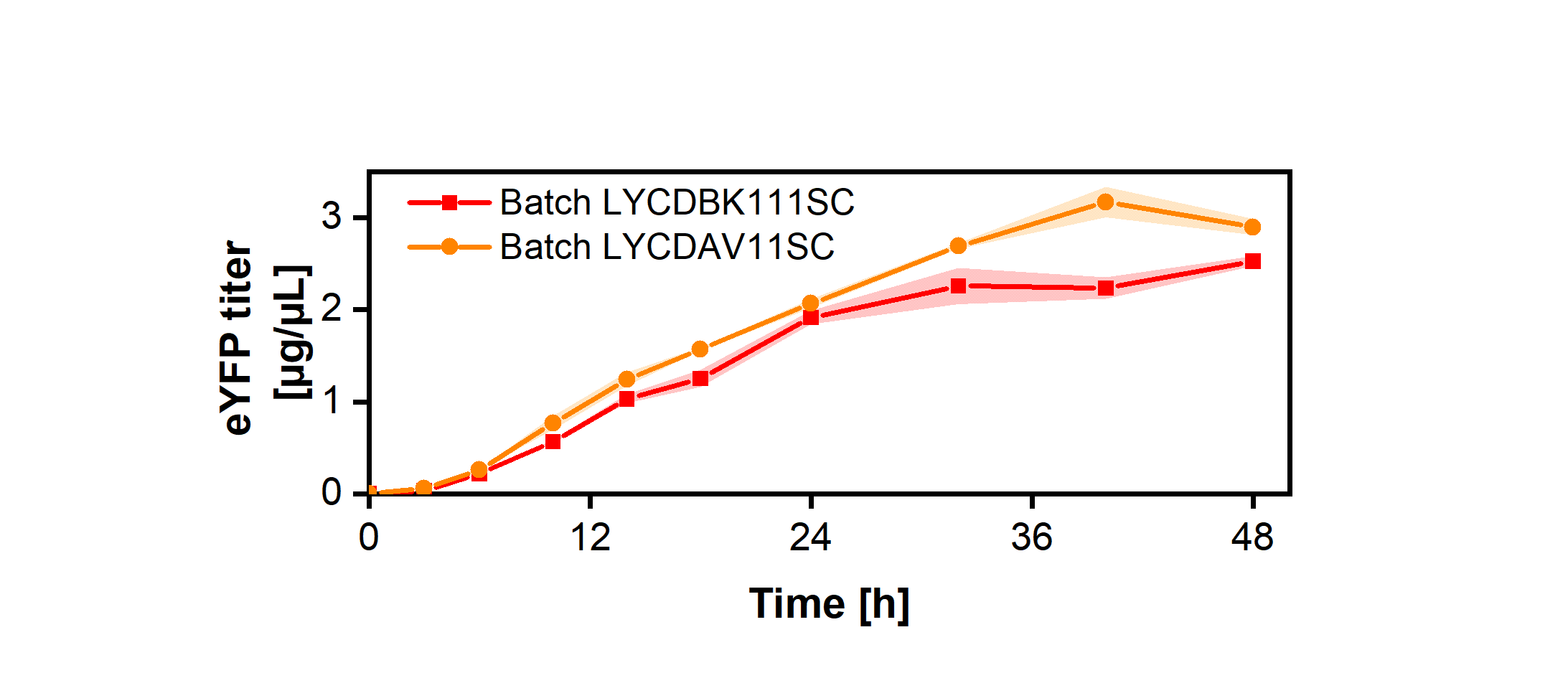


**Figure S2: Comparison of two different lysate reactions at standard conditions.** The lysate reactions were carried out in a 96 half-area plate with 50 µL filling volume per well, operated at 500 rpm, 12.5 mm shaking diameter and 25 °C. Shadows represent the standard deviation of technical quadruple measurements.


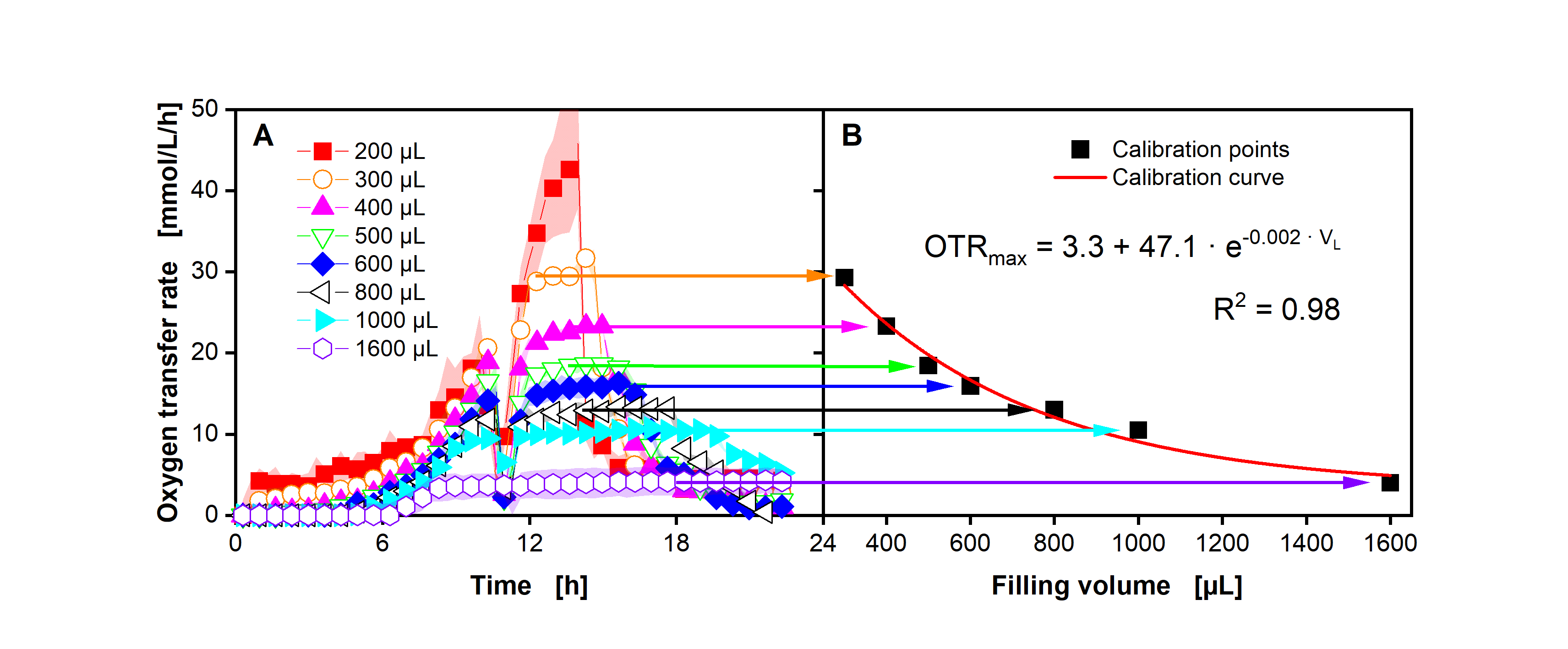


**Figure S3: Calibration curve for the maximum oxygen transfer capacity (OTR_max_), dependent on filling volume in 48-round well microtiter plates.** *Escherichia coli* cultivation in a 48-round well plate with 300 - 1600 µL filling volume per well operated at 700 rpm, 3 mm shaking diameter and 25 °C in a combined µRAMOS-BioLector device (**A**). Colored arrows indicate the OTR_max_ of the corresponding filling volume. The calibration curve is shown in (**B**). The mean of N = 3 replicates is shown (**A**). Shadows represent the standard deviation. For clear data representation, only every second measurement point is shown for the OTR.


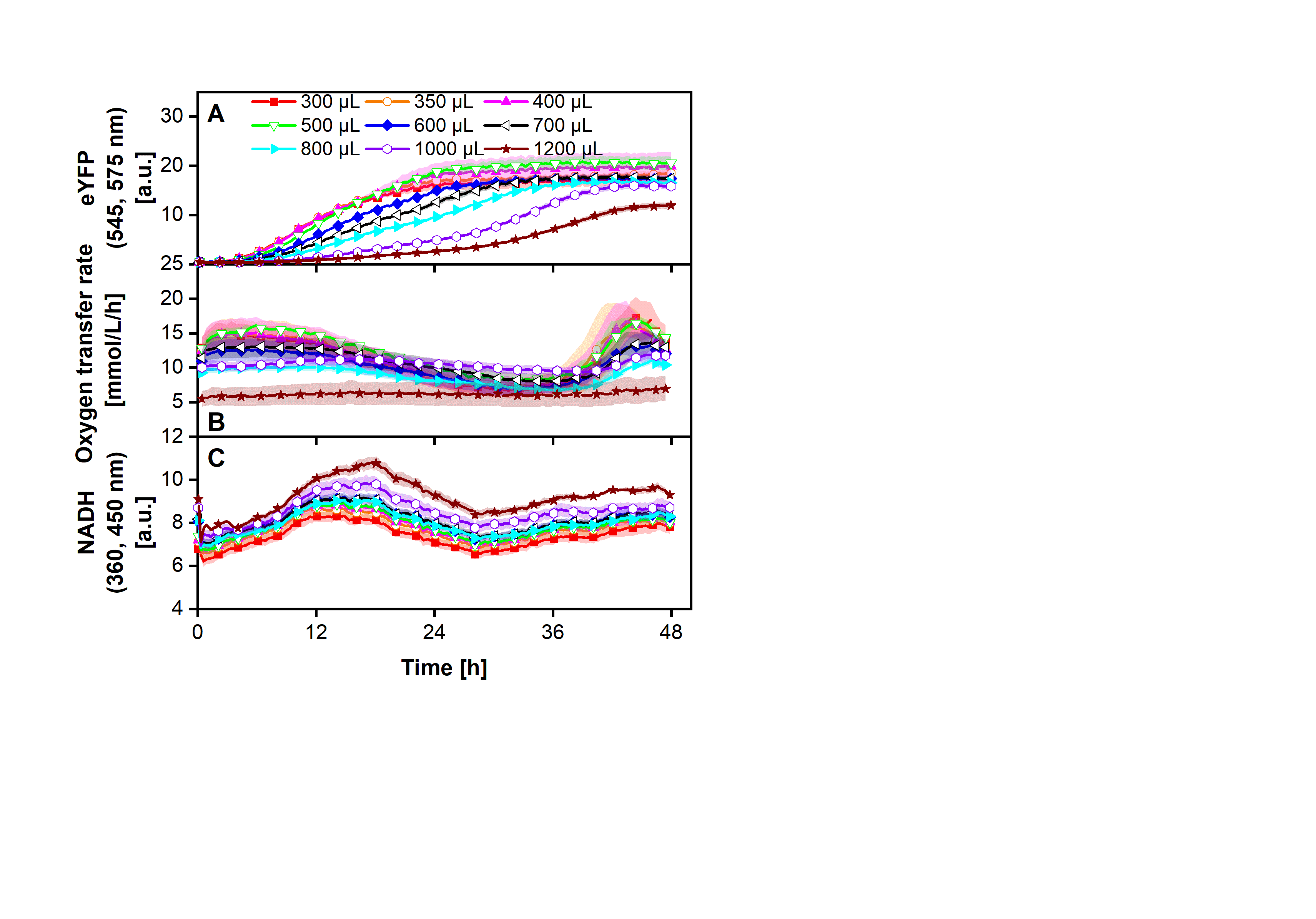


**Figure S4: ALiCE reaction in a 48-round well plate with different filling volumes (including 400 and 500 µL) and online monitoring of the oxygen transfer rate (OTR), the eYFP and NADH fluorescence (compare Figure 2).** The lysate reaction (Batch LYCDBK111SC) was carried out in a 48-round well plate, with 300 - 1200 µL filling volume per well operated at 700 rpm, 3 mm shaking diameter and 25 °C in a combined µRAMOS-BioLector device. The mean of N = 4 replicates is shown. Shadows represent the standard deviation. For clear data representation, only every fourth or eighth measurement point is shown for the OTR and eYFP/NADH, respectively.


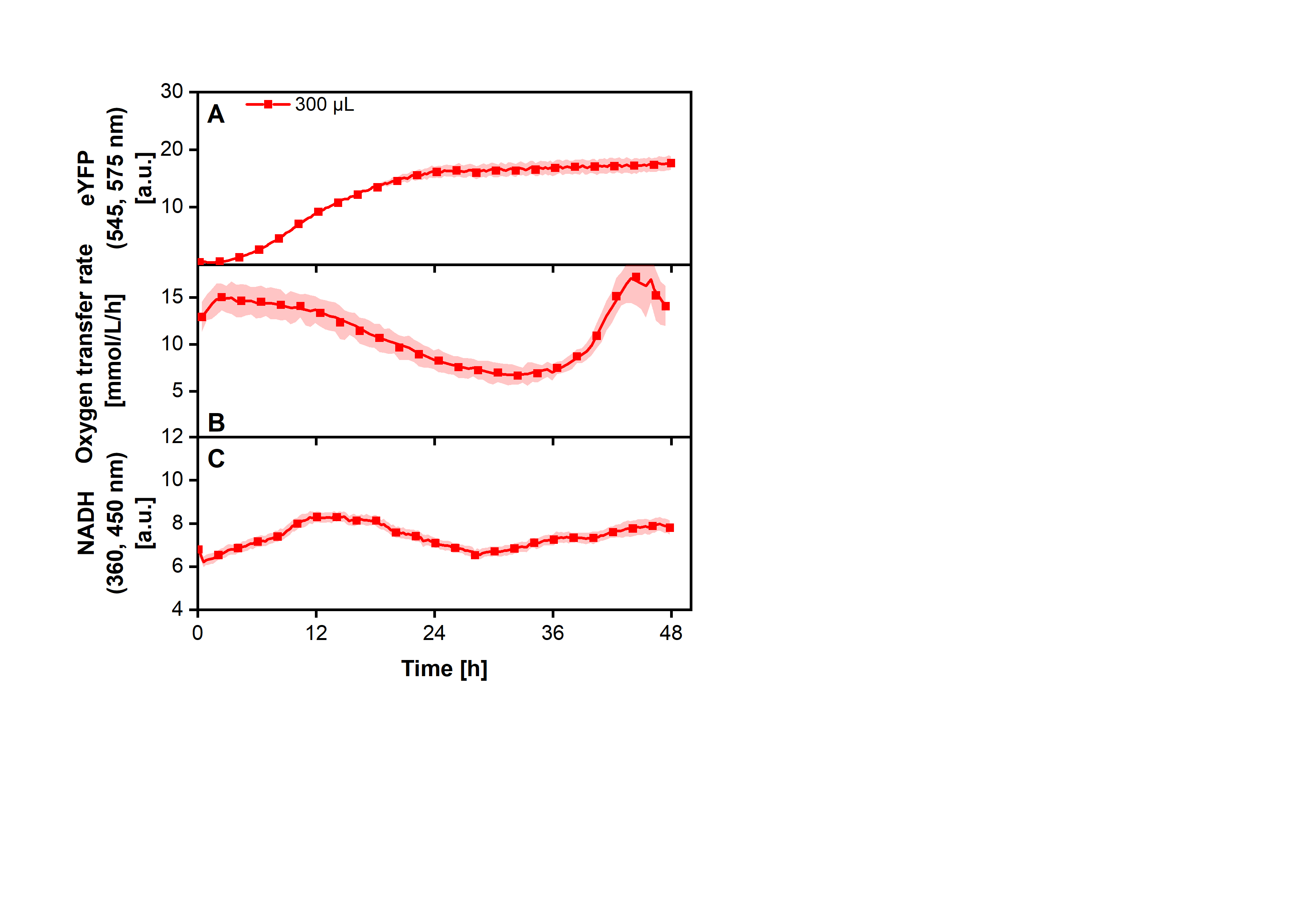


**Figure S5: ALiCE reaction in a 48-round well plate with 300 µL filling volumes (including and online monitoring of the oxygen transfer rate (OTR), the eYFP and NADH fluorescence (compare Figure 2).** The lysate reaction (Batch LYCDBK111SC) was carried out in a 48-round well plate, with 300 µL filling volume per well operated at 700 rpm, 3 mm shaking diameter and 25 °C in a combined µRAMOS-BioLector device. The mean of N = 4 replicates is shown. Shadows represent the standard deviation. For clear data representation, only every fourth or eighth measurement point is shown for the OTR and eYFP/NADH, respectively.


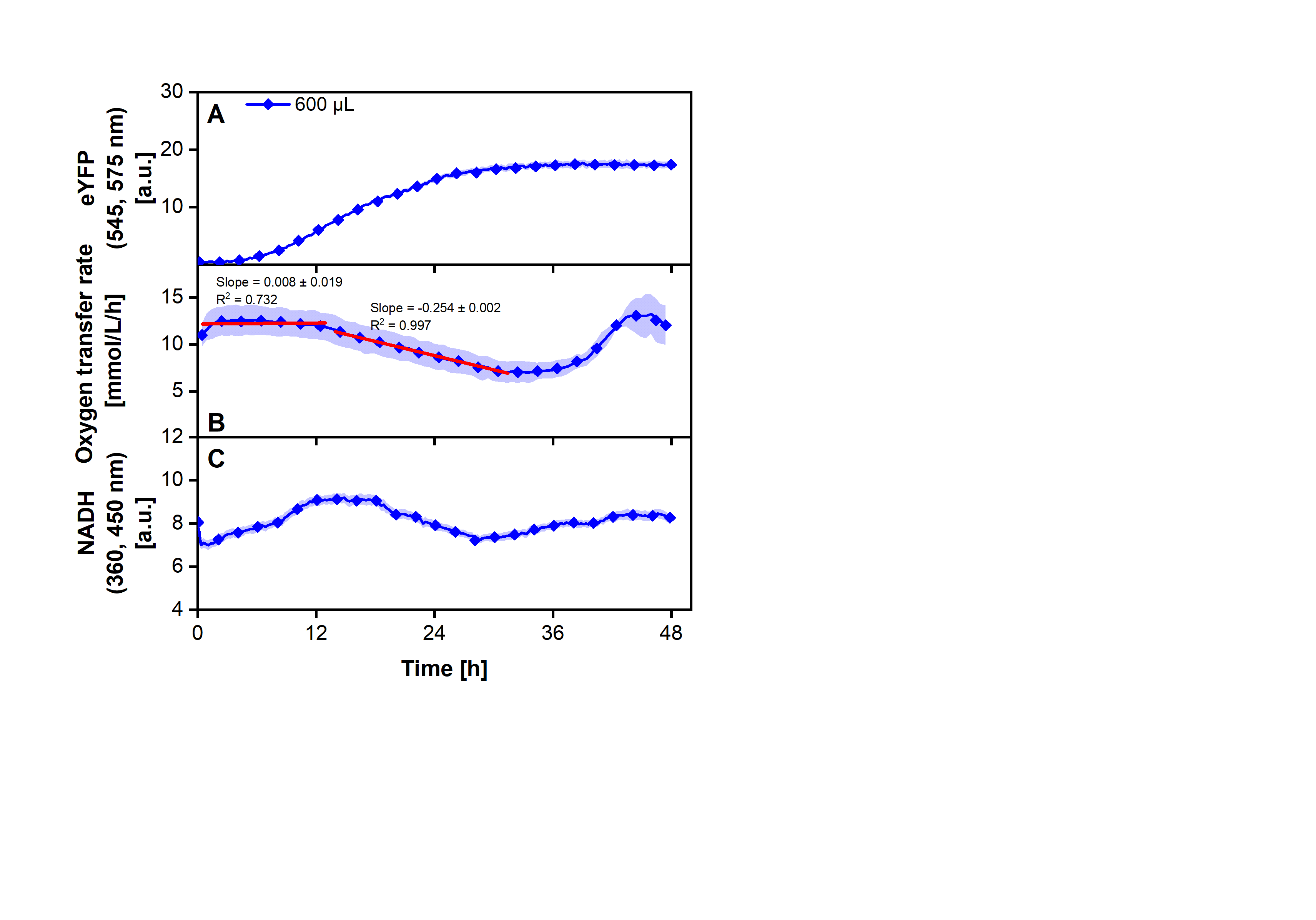


**Figure S6: ALiCE reaction in a 48-round well plate with 600 µL filling volumes (including and online monitoring of the oxygen transfer rate (OTR), the eYFP and NADH fluorescence (compare Figure 2).** The lysate reaction (Batch LYCDBK111SC) was carried out in a 48-round well plate, with 600 µL filling volume per well operated at 700 rpm, 3 mm shaking diameter and 25 °C in a combined µRAMOS-BioLector device. The mean of N = 4 replicates is shown. Shadows represent the standard deviation. For clear data representation, only every fourth or eighth measurement point is shown for the OTR and eYFP/NADH, respectively.


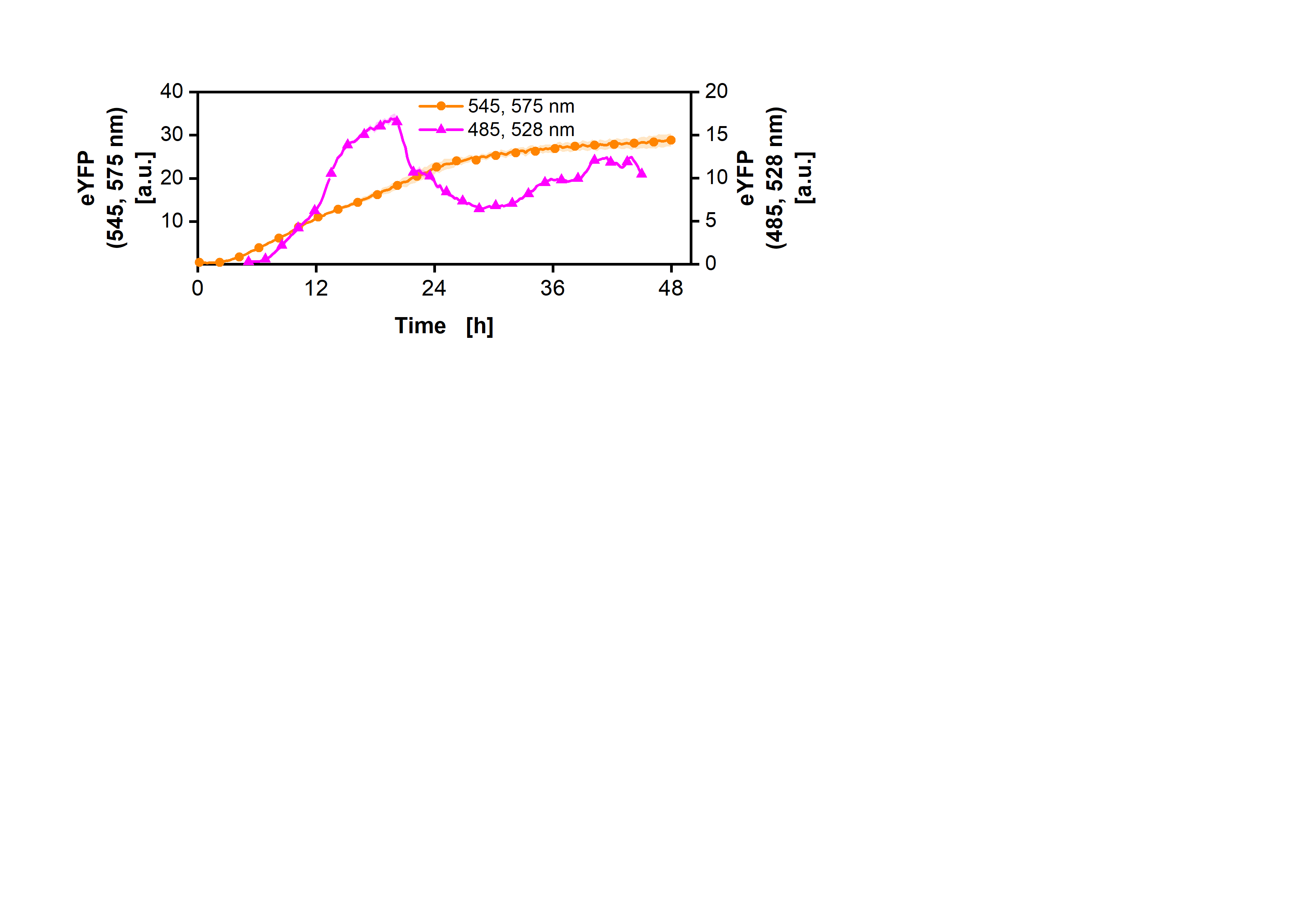


**Figure S7: Comparison of eYFP signals measured with 545/575 nm and 485/528 nm wavelength combination.** The lysate reaction (Batch LYCDAV11SC) was carried out in a 48-round well plate with 300 µL filling volume per well, operated at 700 rpm, 3 mm shaking diameter and 25 °C in a combined µRAMOS-BioLector device. The mean of N = 4 replicates is shown. Shadows represent the standard deviation. For clear data representation, only every eighth measurement point is shown.


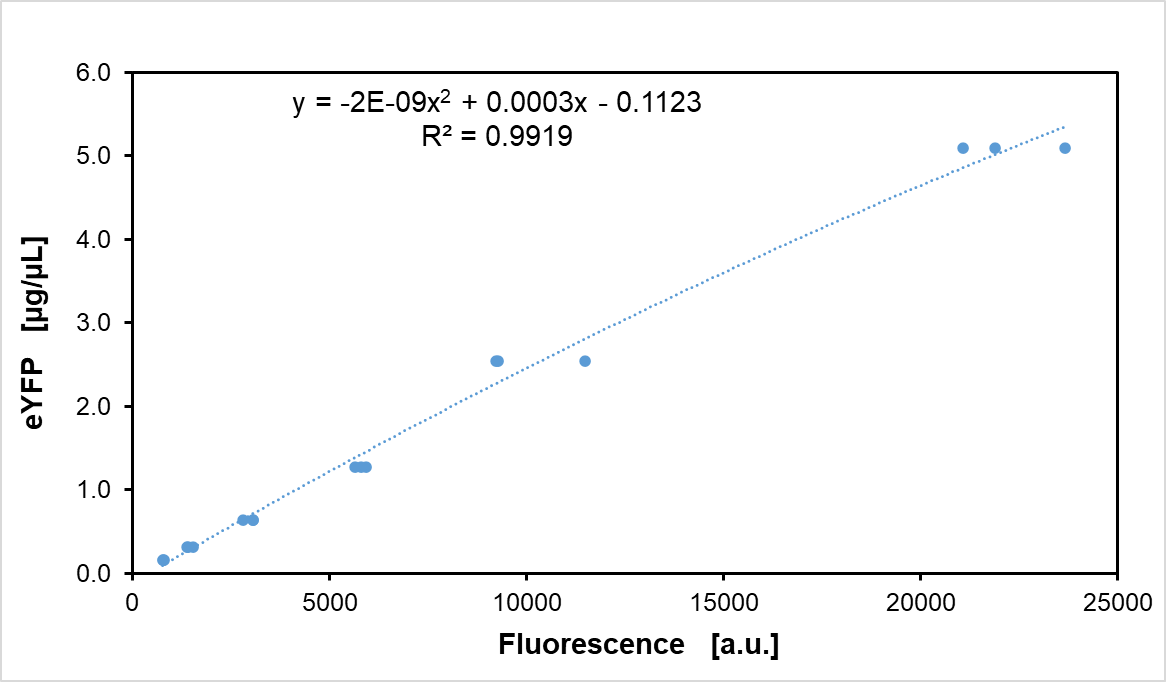


**Figure S8: Exemplary standard curve for offline eYFP determination by fluorescent measurement in a microplate reader.** Samples with known eYFP concentrations (in µg/µL) were measured to generate a standard curve (485, 528 nm).


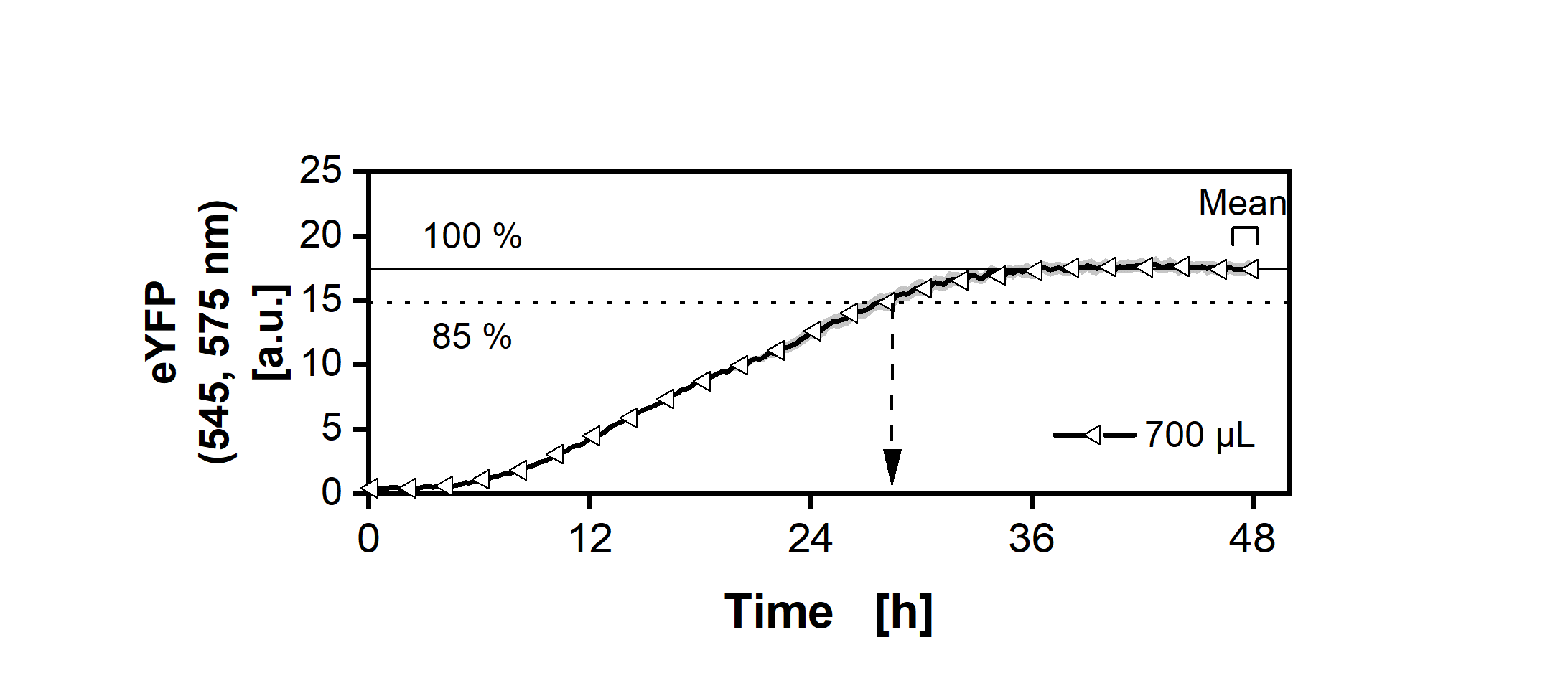


**Figure S9****: Exemplary determination of the mean maximum eYFP signal and the time, at which 85 % of the maximum is reached.** The solid line represents the mean of the last five measurement points (measured over two hours), equal to 100 % of the signal. The dotted line represents 85 % of the maximum. The dashed arrow shows the time at which 85 % of the maximum is reached. The lysate reaction (Batch LYCDBK111SC) was carried out in a 48-round well plate, with 700 µL filling volume per well operated at 700 rpm, 3 mm shaking diameter and 25 °C in a combined µRAMOS-BioLector device. The mean of N = 4 replicates is shown. Shadows represent the standard deviation. For clear data representation, only every eighth measurement point is shown.

**Supplement 10: Derivation of dissolved oxygen tension from OTR and OTR_max_**

The dissolved oxygen tension (DOT) quantifies the oxygen availability in the liquid phase relative to its saturation concentration under the given conditions. The calculation of DOT can be derived from the oxygen transfer rate (OTR), assuming steady-state conditions and first-order mass transfer kinetics. The OTR (mmol/L/h) can be determined using Equation SE1.

The oxygen transfer rate at any given dissolved oxygen tension (DOT) is:

$OTR=k_{L}a \cdot(C^{*}-C)$ (SE1)

with k_L_a as the volumetric mass transfer coefficient (1/h), C* as the saturation concentration of oxygen in the medium (mmol/L) and C as the dissolved oxygen concentration (mmol/L). The maximum oxygen transfer capacity (mmol/L/h), corresponds to the situation where the driving force is at its maximum (i.e. C = 0):

${OTR}_{max}= k_{L}a \cdot C^{*}$ (SE2)

With

$DOT \left[ \% \right]= \frac{C}{C^{*}}\cdot100$ (SE3)

the OTR can be described in dependency of the DOT:

$OTR=k_{L}a \cdot C^{*}\cdot(1-DOT)$ (SE4)

Using SE2 and rearranging for DOT yields equation SE5:

$DOT \left[ \% \right]=100 \cdot(1-\frac{OTR}{{OTR}_{max}})$ (SE5)

Equation SE5 shows that once the measured OTR is below OTR_max_ the DOT will increase and thus, oxygen is available.
